# Supplementary material for: Physicochemical, Rheological, and Sensory Properties of Organic Goat’s and Cow’s Fermented Whey Beverages with Kamchatka Berry, Blackcurrant, and Apple Juices Produced at a Laboratory and Technical Scale
Source: Foods. 2025 Dec 21;15(1):16. doi: 10.3390/foods15010016 (PMC12785497; doi:10.3390/foods15010016)
Supplement: Supplementary file 1 [file foods-15-00016-s001.zip › foods-4004792-supplementary.pdf]

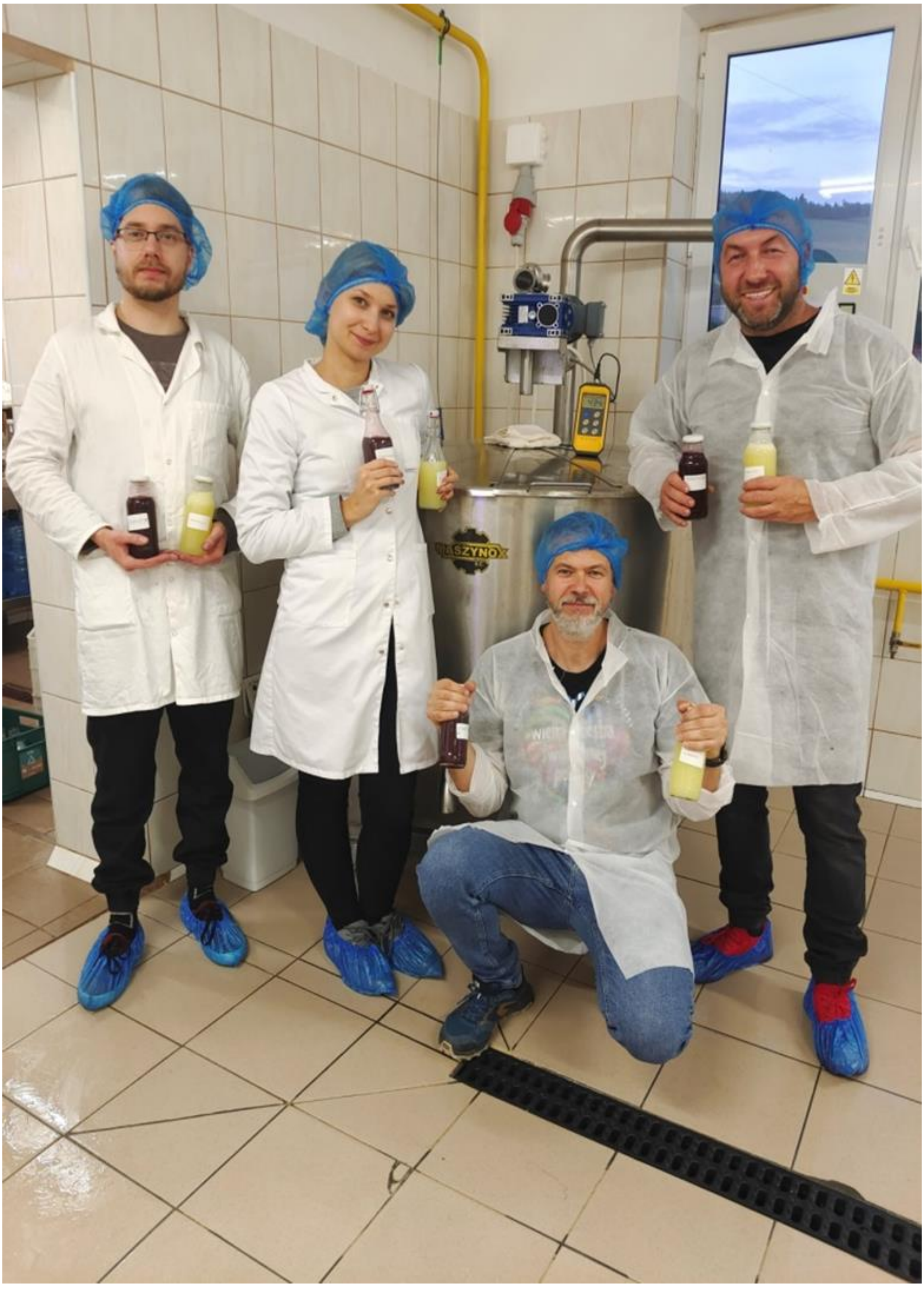

Photo of the Authors and obtained fermented whey beverages in technical scale

Supplementary materia: ANOVA Tables (below)

Table S1. ANOVA for titratable acidity (°SH) of fermented whey beverages.

Source of variation	Sum of squares (SS)	Degrees of freedom (df)	Mean square (MS)	F-value	p-value
Between groups (Zmn1)	13208.73	17	776.98	469.00	<0.0001
Error	29.82	18	1.66	–	–
Total	13238.55	35	–	–	–

p-value < 0.05 was considered statistically significant; p-value ≥ 0.05 was considered non-significant.

Table S2. ANOVA for apparent viscosity of fermented whey beverages.

Source of variation	Sum of squares (SS)	Degrees of freedom (df)	Mean square (MS)	F-value	p-value
Between groups (Zmn1)	85086.28	83	1025.14	35.043	<0.0001
Error	4914.65	168	29.25	–	–
Total	90000.93	251	–	–	–

p-value < 0.05 was considered statistically significant; p-value ≥ 0.05 was considered non-significant.

Table S3. ANOVA for loss modulus G'' of fermented whey beverages.

Source of variation	Sum of squares (SS)	Degrees of freedom (df)	Mean square (MS)	F-value	p-value
Between groups (Zmn1)	17.57930	42	0.41855	304.327	<0.0001
Error	0.57352	417	0.00138	–	–
Total	18.15282	459	–	–	–

p-value < 0.05 was considered statistically significant; p-value ≥ 0.05 was considered non-significant.

Table S4. ANOVA for storage modulus G' of fermented whey beverages.

Source of variation	Sum of squares (SS)	Degrees of freedom (df)	Mean square (MS)	F-value	p-value
Between groups (Zmn1)	112.8542	42	2.6870	1109.81	<0.0001
Error	1.0096	417	0.0024	–	–
Total	113.8638	459	–	–	–

p-value < 0.05 was considered statistically significant; p-value ≥ 0.05 was considered non-significant.

Table S5. ANOVA for pH

**Source of variation**	**Sum of squares (SS)**	**Degrees of freedom (df)**	**Mean square (MS)**	**F-value**	**p-value**
Between groups (Zmn1)	27.113	41	0.661	2374	<0.0001
Error	0.023	84	0.000	–	–
Total	27.136	125	–	–	–

p-value < 0.05 was considered statistically significant; p-value ≥ 0.05 was considered non-significant.
